# The molecular mechanism of the adverse effects of the biological and small molecular drugs in the therapy of inflammatory skin diseases – psoriasis and atopic dermatitis

**DOI:** 10.1080/07853890.2025.2611461

**Published:** 2026-01-02

**Authors:** Patrycja Lemiesz, Julia Nowowiejska-Purpurowicz, Iwona Flisiak

**Affiliations:** Department of Dermatology and Venereology, Medical University of Bialystok, Bialystok, Poland

**Keywords:** Atopic dermatitis, psoriasis, dupilumab, upadacitinib, conjunctivitis, TNFα inhibitors, JAK inhibitor

## Abstract

Patients with the most common chronic inflammatory dermatoses, namely psoriasis and atopic dermatitis, gained access to state-of-the-art therapeutic options providing spectacular improvement of skin lesions. Although generally safe, biological agents and small molecular drugs have also side effects which may be mild and irrelevant to the therapy course, but sometimes, of a greater extent and influencing further therapeutic decisions. In this review, we summarize the molecular explanation for the most common adverse effects of drugs used in the treatment of psoriasis and atopic dermatitis. Biologics used in psoriasis predominantly target TNFα, IL-17, 23, while in AD inhibit IL-4,13,31. Janus kinase (JAK) inhibitors represent small-molecule therapies effective in both conditions, although more prominently in AD. TNFα inhibitors are associated with increased susceptibility to infections, paradoxical psoriasis, eczematoid lesions, and reactivation of tuberculosis. IL-17 inhibitors may cause fungal infections and possibly trigger inflammatory bowel disease. IL-4/13 blockade in AD treatment has been linked to eosinophilia, conjunctivitis, arthritis, and facial erythema, likely due to a shift toward IL-17-driven inflammation. JAK inhibitors may cause infections, acne, dyslipidemia, and, in selected cases, cardiovascular events. This review emphasizes the importance of understanding the pathogenetic background of drug-related complications to introduce appropriate clinical management and patient selection.

## Introduction

Psoriasis is a chronic, incurable, inflammatory skin disease that affects an average of 3% of the global population [1]. It is one of the most common reasons for appointments in the dermatological office. The pathogenesis of psoriasis is complex and still not fully elucidated; it encompasses genetic background, immunological disorders, and the influence of modulating environmental factors [2,3]. Currently, more than 60 chromosomal *loci* determining predisposition to the disease have been identified [2]. Psoriasis is also undoubtedly an autoimmune disease, which can be provoked and exacerbated by external factors, e.g. trauma, infection, stress, and drugs [1,2]. Clinically, the most common variant of psoriasis – plaque – is manifested by the presence of papules which merge forming exfoliative psoriatic plaques in upright locations of the extremities, lumbar and sacral regions, and in the intragluteal fold. The diagnosis is usually made based on the clinical picture [1,3].

Atopic dermatitis (AD) is also one of the most common chronic skin diseases. As with psoriasis, the pathogenesis of AD is complex and includes genetic background, immunological disorders, and modulating environmental factors [4]. The essence of AD is epidermal barrier dysfunction, resulting in water loss and subsequent severe dryness, which in turn leads to easier penetration of pathogens and allergens [4]. The clinical picture depends on the age of the patient; adults have erythematous and exfoliative lesions with lichenification on the flexor surfaces of the extremities, while in children the lesions are more exudative in nature and have the opposite localization – on the upright surfaces of the extremities, or on the face. The skin lesions are accompanied by pruritus [4]. It should also be mentioned that AD is a heterogeneous disease, and there are significant racial differences in the pathogenesis and observed clinical picture. The diagnosis is usually made based on the Hanifin and Rajka criteria [5].

Currently, both psoriasis and AD patients have the possibility of modern treatment with biological and small-molecular drugs.

**Figure 1.**
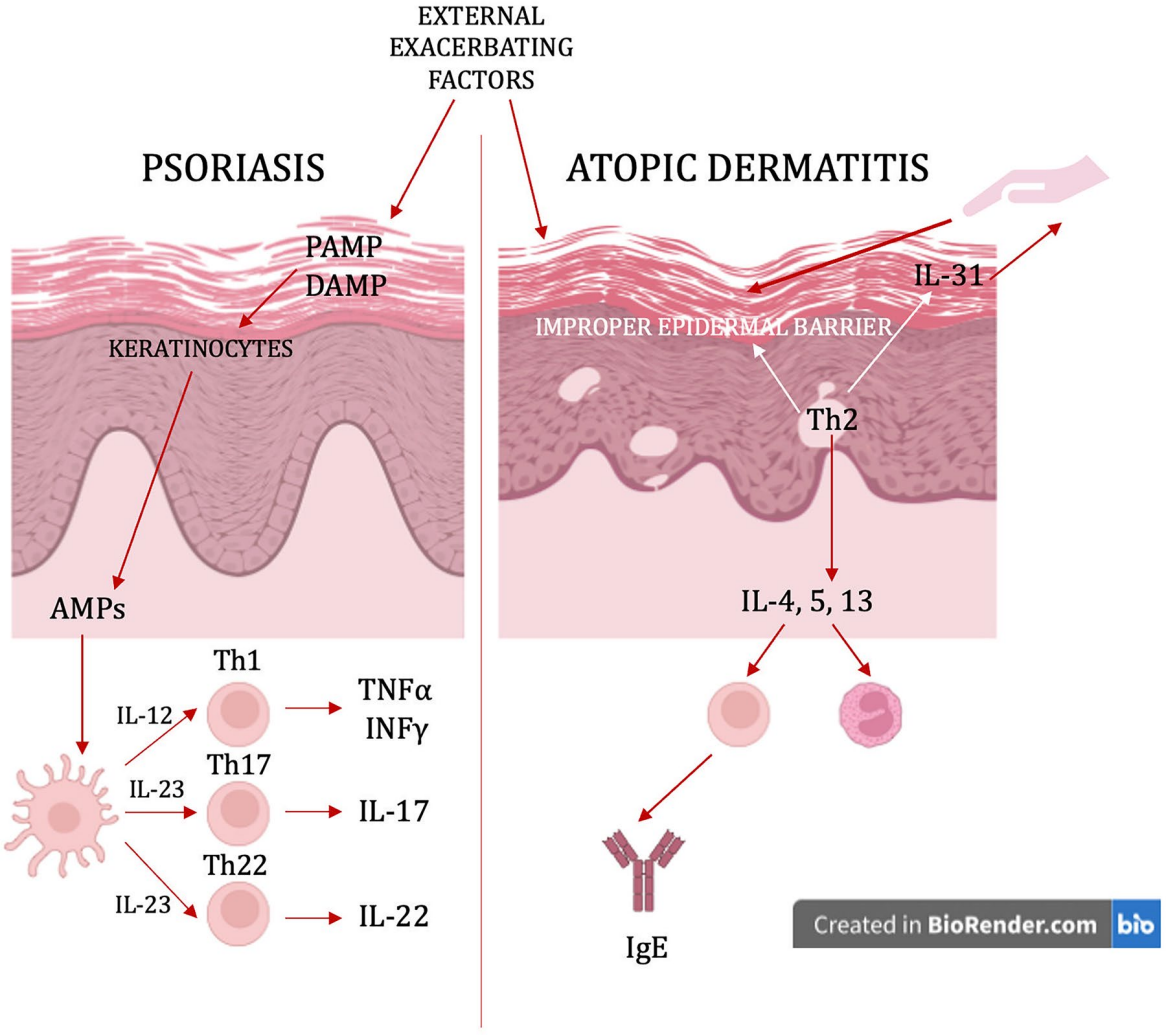
The summary of pathogenesis and comparison between psoriasis and atopic dermatitis. On the left – psoriasis: after the exposure to environmental stimuli, damage-related molecular patterns (DAMPs) and pathogen-associated molecular patterns (PAMPs) appear in genetically predisposed persons and stimulate keratinocytes to release antimicrobial peptides (AMPs), which further activate dendritic cells (DCs). They secrete several cytokines: IL-23 stimulates the differentiation of naïve T cells into the Th17 and Th22 subpopulation, IL-12 promotes the Th1 line. Th17 lymphocytes secrete IL-17, Th22 cells secrete IL-22, Th1 cells secrete TNF and IFN. On the right – atopic dermatitis (AD): the hallmark of AD is an improper epidermal barrier, which leads to loss of water followed by skin dryness, itch and scratching. Moreover, a disrupted barrier enables pathogens and allergens to penetrate, which leads to disease exacerbations. Th2 lymphocytes secrete IL-4, IL-5 and IL-13. IL-4 increases the number of eosinophils, the synthesis of IgE antibodies, leads to decreased AMP and filaggrin expression. IL-4 can inhibit Th1 and Th17 and stimulate Th2, directly influencing T lymphocytes. Th2 may also secrete IL-31, which is related to pruritus and promotes scratching.

Biological treatment involves the use of monoclonal antibodies or their fragments, which target specific cytokines or their receptors, which are engaged in the pathogenesis of particular diseases [6]. Monoclonal antibodies are big molecules, which results in their route of administration as they are administered as subcutaneous injections. The main cytokines involved in the pathogenesis of psoriasis are certainly TNFα, IL-12, IL-17, and IL-23 [2], whereas in the pathogenesis of AD, IL-4, IL-13, and IL-31 [7] (Figure 1).

Janus kinase inhibitors (JAK inhibitors) are a recent type of therapeutics that act by interfering with cytokine signaling through the Janus kinase-signal transducer and activator of transcription (JAK-STAT) pathway. Therefore, they affect immune responses and cell growth. So far, four members of the JAK family kinases have been distinguished (JAK1-3 and tyrosine kinase 2 – TYK2). JAK inhibitors act by targeting the kinase part of the JAK, blocking its ability to phosphorylate. This interruption stops the intracellular signaling process from continuing [8]. JAK1 is associated among others with IL-2,4,10, JAK2 with IL-3,5,12,23, JAK3 with IL-2,4, TYK2 with IL-10,12,22,23 [9]. JAK inhibitors are small molecules; hence, they can be administered orally.

To the best of our knowledge, this is the first review that summarizes the most typical adverse effects of the biological and small molecular treatment in the most frequent inflammatory skin condition, providing the pathogenetic background for these complications.

## Biological and small molecular drugs used in the therapy of psoriasis

TNFα antagonists group includes infliximab, adalimumab, certolizumab pegol, which are monoclonal antibodies against TNFα, and etanercept, a TNFα receptor fusion protein, binding to soluble TNFα. Etanercept received its first United States Food and Drug Administration (FDA) approval in the treatment of adult patients with plaque psoriasis in 2004, infliximab in 2006, adalimumab in 2008, and certolizumab pegol in 2018. In 2016, the FDA allowed etanercept to be administered to children over the age of 4 [10–13]. Adalimumab was approved for psoriasis in children aged 4 and older by the European Medicines Agency (EMA) in 2015, while it did not gain registration in pediatric patients in the United States [14].

Ustekinumab is the only representative of IL-12 and 23 inhibitors approved by the FDA for the treatment of psoriasis in adults in 2009; as of 2017, the indication has been extended to children aged 12 and older, and as of 2020, to patients aged 6–11 as well [15].

The IL-17 antagonists currently registered for the treatment of psoriasis are secukinumab, ixekizumab, brodalumab and bimekizumab. Secukinumab, an IL17A blocker, received FDA approval in adult plaque psoriasis in 2015, and in 2012 in children over 6 years of age [16]. Ixekizumab, also an IL-17A-neutralizing agent, was approved by the FDA for the treatment of psoriasis in adults in 2016, and in 2020 in people aged 6–18 [17]. Brodalumab, a monoclonal antibody binding to IL-17 receptor A and blocking IL-17A, IL-17C, IL-17F, IL-17A/F, and IL-17E (also known as IL-25), got the FDA approval for adult psoriasis in 2017 [18]. Bimekizumab, the group’s newest representative, which blocks IL-17A and IL-17F, received FDA approval for the treatment of psoriasis in adults in 2023 [19].

Representatives of IL-23 antagonists approved by the FDA for the treatment of psoriasis in adults are guselkumab (since 2017), tildrakizumab (2018), and risankizumab (2019) [20–22].

The only small-molecule drug approved by the FDA in 2022 for the treatment of psoriasis in adults is deucravacitinib, which inhibits a tyrosine kinase 2 (TYK2) mediating IL-23, IL-12, and IFN type I signaling [23].

Table 1 summarizes the modern drugs available in the therapy of psoriasis (Table 1).

**Table 1.** The summary of biological and small-molecular drugs used in the treatment of psoriasis and atopic dermatitis and their most typical side effects.

Biological drugs				Small molecules		
Psoriasis		Atopic dermatitis		Psoriasis	Atopic dermatitis	
Adalimumab	Infections Paradoxical psoriasis TNFα inhibitor-associated psoriatic alopecia Eczematous reactions	Dupilumab	Conjunctivitis Eosinophilia Facial erythema Lymphoma-toid reactions Parasitic infections	Deucravacitinib	Baricitinib	Dyslipidemia and weight gain Acne Thrombo-embolic complications Neoplasms
Etanercept		Tralokinumab	Conjunctivits Eosinophilia		Upadacitinib	
Certolizumab pegol		Lebrikizumab	Parasitic infections?		Abrocitinib	
Infliximab		Nemolizumab	Potential exacerbation of preexisting asthma			
Ixekizumab	Inflammatory bowel disease Infections, particularly fungal					
Secukinumab						
Bimekizumab						
Brodalumab						
Guselkumab	Infections					
Tildrakizumab						
Risankizumab						

Yellow means TNF inhibitors, green means IL17 inhibitors, blue means IL23 inhibitors, pink means IL4/13 inhibitor, orange means IL13 inhibitors, grey means IL31 inhibitor, red means JAK1 inhibitors, purple means JAK1/2 inhibitor, white means TYK inhibitor.

## Biological and small molecular drugs used in the therapy of atopic dermatitis

Dupilumab is a monoclonal antibody blocking the IL-4 receptor, hence inhibiting both IL-4 and IL-13. First FDA approval of dupilumab for AD took place in 2019, but then, in 2022, it was approved for patients as young as 6 months of age [24].

Tralokinumab is a monoclonal antibody blocking IL-13. It was first approved for AD by the FDA in 2021, and in 2023, it was approved for use in patients as young as 12 years old [25].

Lebrikizumab is the newly approved drug for AD, in September 2024. It is a monoclonal antibody blocking IL-13 [26].

Nemolizumab is also the newly approved drug for AD, in December 2024. It is a monoclonal antibody blocking IL-31 receptor, approved for AD in patients as young as 12 years old [27].

Upadacitinib is a JAK1 inhibitor. It was approved for AD by the FDA in 2022 for patients as young as 12 years old [28].

Baricitinib is a JAK1/2 inhibitor. It was approved for the treatment of AD for patients as young as 2 years old only in Europe. It is not approved by the FDA [29].

Abrocitinib is a JAK1 inhibitor. It was first approved by the FDA in adult patients with AD in 2022, but in 2023, in patients from 12 years old [30].

Table 1 summarizes the drugs available in the therapy of AD (Table 1).

## Adverse effects of the biological and small molecular drugs used in the therapy of psoriasis

Currently available biologic drugs for the treatment of psoriasis target TNFα, IL-17, and IL-23. Under inflammatory conditions, TNFα is produced by various cells, both of the immune system (such as macrophages, Th1, Th17, Th22 lymphocytes) and by keratinocytes, and its serum levels correlate with psoriasis severity [31]. Stimulation of TNFα receptors results in the formation of signaling complexes – nuclear factor κB (NF-κB), mitogen-activated protein kinases (MAPKs), caspase-8, mixed lineage kinase domain-like protein (MLKL) – inducing inflammation, cell survival and proliferation, immune response against pathogens, apoptosis, necroptosis and inflammation [32]. TNFα activates dendritic cells (DC) and IL-12 secretion, leading to differentiation of naïve T cells into the Th1 phenotype. Th1 cells secrete more TNFα, resulting in aggravation of inflammation. IL-23, a proinflammatory cytokine belonging to the IL-12 family, produced particularly by DC, induces differentiation of Th17 cells – the main source of IL-17, which comes in six isoforms (IL-17A-F). IL-17, through IL-19, promotes keratinocyte migration and keratinocyte growth factor (KFG) production by fibroblasts [33]. Moreover, IL-17 activates secretion of multiple cytokines, chemokines, and antimicrobial peptides (AMP) and proteins by keratinocytes, fibroblasts, and other innate immune cells [34].

### All biological agents

The most common adverse effects associated with the use of biological drugs in psoriasis, regardless of their group, observed both in clinical trials and in real-world evidence data, are undoubtedly infections [35–39]. According to a study using data from BIOBADADERM, a multicenter, prospective cohort study comparing the risk of infection during the use of biological drugs (from the anti-TNF group and ustekinumab) to classic drugs used in psoriasis, it was significantly increased for TNF antagonists [40]. A nationwide cohort study from France conducted by Penso et al. showed that the risk of severe infection, defined as any infection leading to hospitalization, was higher for new users of infliximab and adalimumab compared to etanercept, lower for ustekinumab, and without increased risk for IL-17 and 23 inhibitors vs etanercept [41]. TNFα is crucial for antiviral immunity, activating immune cells such as macrophages, NK cells, and T cells [42]. For this reason, it is not surprising that the use of TNFα inhibitors also carries a higher risk of hepatitis B and C reactivation compared to anti-IL17 and 23 agents, and therefore, drugs from the latter two groups should be considered as a therapeutic option in concurrent HBV or HCV infection [43].

Safety concerns regarding malignant neoplasms have been raised, especially in subjects with a history of cancer in the last 5 years [44]. Such reflections arise from the fact that patients with psoriasis are thought to be at increased risk of malignancies, and the biological agents may target some molecules that affect anti-cancer immune response (particularly TNFα) [44]. In recent years, there have been more and more reports on the successful introduction of biological agents in psoriasis with no malignancy recurrence [44]. This issue is, however, controversial, and although the approach has become more liberal, every case should be treated individually and consulted with an oncologist. The risk of malignancy seems to be very low in subjects treated with IL-17 or IL-23 inhibitors [45]. More doubts concern anti-TNFα agents, especially the development of non-melanoma skin cancers; however, the risk is associated with many additional factors, including age, the previous history of immunosuppressive therapy, or hematological disorders [45].

### Biological agents – TNFα inhibitors

TNFα antagonists are the longest-used biologic agents in the treatment of psoriasis; they are also widely used in the treatment of other immune-mediated diseases, such as psoriatic (PsA) and other seronegative arthropathies (SA), rheumatic arthritis (RA), inflammatory bowel diseases (IBD), hidradenitis suppurativa (HS), or uveitis, in some indications also in children [46].

#### Tuberculosis

Another well-known side effect of anti-TNFα drugs is an increased risk of tuberculosis (TB) development. According to research, the risk is higher for monoclonal antibodies (infliximab, adalimumab) than soluble receptor (etanercept) [47–50]. The incidence of TB associated with TNFα inhibitor use is higher in South America and Asia than in North America and Europe, thus it correlates with the incidence of TB in the general population [51]. It can be either reactivation of latent TB or a new infection acquired during follow-up [48]. Accordingly, international guidelines recommend TB screening and prophylactic treatment for latent TB in patients expected to receive anti-TNFα drugs [52]. Immunity against *Mycobacterium tuberculosis* (Mtb) is mediated by antigen-specific T cells – CD4+, secreting INFγ, and CD8+, releasing Th1 cytokines [53], including TNFα, which is known to have an essential role in the formation of limiting Mtb growth granulomas *via* macrophage activation and mediates cell death through caspase-dependent apoptotic efflux [54]. As shown in the study by Hamdi et al. TNFα antagonists *in vivo* reduce the population of IFN-releasing memory CD4+ T cells after stimulation by Mtb, both antibodies and soluble TNFα receptor, while *in vitro,* etanercept was less effective in inhibiting activation of CD4+ T cells by Mtb antigens [55]. In addition, in a mouse model, anti-TNFα antibodies showed better penetration than the receptor for TNFα into granulomas in chronic TB, which may correspond to less neutralization of this cytokine by etanercept [56]. Due to impaired IFNγ secretion in individuals treated with TNFα antagonists, tests based on IFNγ release should not be used in these patients for the diagnosis of TB [55].

Regarding the use of IL-17 and IL-23 inhibitors and TB, evidence to date indicates that they do not increase the risk of TB reactivation, and these two interleukins are probably not essential in the pathogenesis of immune response against Mtb [57].

#### Paradoxical psoriasis

Even though this group of biological drugs is an important therapeutic option for psoriasis, their ability to cause paradoxical psoriasis (PP) is known [58]. PP can manifest as both de novo psoriasis and exacerbation of already existing skin lesions, and as any form of psoriasis; the most described are plaque psoriasis (15.8–50%) and palmoplantar pustular psoriasis (33.3–45%); the scalp and nails can also be involved [59,60]. Although PP was previously thought to be an adverse effect of an entire group of drugs [61], according to a follow-up study by Yagiz et al. switching to another TNFα antagonist can result in partial or complete remission of skin lesions [62]. Interestingly, the incidence of PP varies depending on the condition being treated; for instance, in ankylosing spondylitis, it is determined at 0.5–1% [58] and at almost 5% in IBD [63]. Classical psoriasis is mediated by autoimmune T-cells, stimulated by plasmacytoid dendritic cells (pDCs)-derived type 1 interferons [64]. PP development may be the result of cytokine imbalance after TNFα blocking. TNFα inhibits the maturation of pDCs from progenitor cells, causing suppression of IFNα production, so TNFα blocking will result in increased IFNα production by pDCs [63,65,66]. IFNα plays an important role in the induction of psoriatic lesions – the IFN signaling pathway is activated in psoriatic skin, psoriatic T cells are sensitive to IFN, furthermore, IFN induces the expression of CXCR3 on T cells, making it easier for them to reach the skin [67–69]. De Gannes et al. showed increased type 1 IFN in skin biopsies in TNFα inhibitor-induced psoriasis compared to psoriasis vulgaris [66]. Histological analysis by Tillack et al. revealed an increased number of IFNγ-secreting Th1 and IL-17-/IL-22-secreting Th17 cells in anti-TNF-induced psoriasiform skin lesions [63]. Apart from IL-17, IL-22 has a crucial role in the pathogenesis of psoriasis, mediating IL-23-induced acanthosis and dermal inflammation through the activation of STAT3 (signal transduction and activators of transcription 3) *in vivo* [70]. The management of PP depends on the severity of skin lesions and control of the underlying disease. Li et al. proposed an algorithm for the management of anti-TNF-induced PP [59].

#### TNFα inhibitor-associated psoriatic alopecia

TNFα inhibitor-associated psoriatic alopecia (TiAPA) is an infrequent complication of treatment with infliximab and adalimumab in particular, and surprisingly is more common in subjects with IBD than those treated for psoriasis or PsA. It occurs months or years after the therapy introduction and is probably related to IFNα secretion [71]. Usually, the topical agents are sufficient, and discontinuing the culprit drug may not be necessary [72].

#### Eczematous reactions

Eczematous reactions are another common side effect of TNF-α inhibitors, affecting approximately 5 to 20% patients undergoing therapy, and the risk appears to be increased if there is a personal history of atopy. This could be either a new onset of eczema or an exacerbation of previously present lesions [73]. It seems that the infliximab causes eczematous reactions more often than other anti-TNF-α drugs – perhaps due to its greater effectiveness in treating psoriasis (80% of patients achieving 75% or greater improvement in the psoriasis severity and area index -PASI-75- vs. 52% for etanercept, and 59% for adalimumab [74]) and in connection with more effective and rapid Th1 pathway blocking. It is suspected that inhibition of the Th1 response shifts the balance toward the Th2 response, which plays an important role in eczematous diseases [73]. A study by Stoffel et al. showed that eczematous anti-TNF-α skin lesions contained more Th2-related cytokines (IL-13, CCL26, IL-5) compared to psoriasis-like lesions. These cytokines are relevant mediators in eczema [75].

### Biological agents – IL-17 inhibitors

#### Inflammatory bowel disease

IL-17 possesses a few important properties regarding human health. One of them is the influence on the intestinal barrier. However conflicting, the majority of published papers report the protective role of IL-17 on colitis models [76]. IL-17 is considered crucial for the maintenance of the intestinal barrier, probably due to the ability of IL-17A to strengthen the tight junctions between intestinal epithelial cells [77]. Thus, when blocking IL-17, it is possible to cause disintegration of the barrier, and such cases have been widely reported. A more detailed alleged mechanism is that the occludin protein diffuses, leading to increased intestinal permeability and pathogen invasion [77]. At the same time, it must be highlighted that patients with psoriasis have been proven to be at increased risk of IBD (1.70–2.53-fold increased risk of developing Crohn’s disease, and 1.75 times increased risk of developing ulcerative colitis) [78]. Nevertheless, it remains uncertain whether IL-17 inhibitors indeed induce the onset of IBD or just reveal the disease in susceptible individuals, or it may be just a coincidence [77]. There has been a very engaging study that aimed at assessing the characteristics of IBD cases in subjects on IL-17 inhibitors treated for different reasons, including especially psoriasis and arthritis. However, it has to be mentioned that the analysis concerned only secukinumab, ixekizumab and brodalumab; bimekizumab was not included back then. The majority of reported cases involved secukinumab and occurred more frequently in women. The proportion of Crohn’s disease to ulcerative colitis was similar, with a slight advantage of Crohn’s. The authors revealed that among all IBD-induced cases, 91.2% of them had IBD for the first time, and about half of these cases were diagnosed within 3 months of starting the therapy with IL-17 inhibitors. As for the prognosis, the discontinuation of IL-17 inhibitors in combination with corticosteroids or/and TNFα inhibitors resulted in remission [77].

#### Fungal infections

In consideration of the important effect of IL-17 on mucocutaneous antifungal immunity, it is not surprising that its blockade will result in a higher risk of developing fungal infections, especially candidiasis [79–81]. Mutations in genes for IL-17 subunits or their receptors are associated with chronic mucocutaneous candidiasis (CMC), similarly to mutations affecting STAT3 and DOCK8 proteins, resulting in impaired differentiation of IL-17-secreting Th17 cells [82].

Randomized controlled trials comparing secukinumab, etanercept and placebo in psoriasis, as well as in psoriatic arthritis and ankylosing spondylitis, showed a higher incidence of *Candida* infections in the secukinumab arm; these infections resolved spontaneously or with standard antifungal treatment and did not require discontinuation of the drug. Similar data come from a combined study comparing different doses of ixekizumab with etanercept and placebo, showing a significantly higher incidence of superficial candidiasis in the group of patients receiving ixekizumab every 2 weeks than every 4 weeks, as well as etanercept and placebo [83]. Systematic reviews and meta-analyses estimate the incidence of candidiasis as 1.7%, 3.3%, 4.0%, and as high as 6–21.1% for secukinumab, ixekizumab, brodadumab, and bimekizumab, respectively [84].

### Biological agents – IL-23 inhibitors

According to the summary of product characteristics of guselkumab, tildrakizumab, and risankizumab, the most frequent adverse effect is infections (similar to all other biologics), particularly of the respiratory tract [85–87]. To date, there is no information about the side effects characteristic of this group of drugs.

### Deucravacitinib

The most common side effects of JAK inhibitors as a group are described below, as they are more often used in the therapy of AD. As deucravacitinib is a TYK inhibitor, it is not characterized by side effects, such as hyperlipidemia, typical of other JAK inhibitors [88]. Since it is a relatively new drug, further information will come. However, the latest real-world evidence data confirm the favorable safety profile of the drug, which does not differ from the data from clinical trials. Due to the inhibition of the type I IFN pathway by blocking TYK2, which plays a role in antimicrobial and anticancer processes, it will be necessary to assess the risk of malignant tumors and infections in the long-term use of deucravacitinib [89].

## Adverse effects of the biological and small molecular drugs used in the therapy of atopic dermatitis

### Biological agents

Among the biological agents available in the treatment of AD, they are mainly, but not solely, focused on blocking IL-4 and IL-13.

IL-4 and IL-13 are secreted by innate lymphoid cells (ILCs) and Th2 lymphocytes, their synthesis is promoted by TSLP, and NFκB and MAP is involved in the pathogenesis od AD in several ways. IL-4 increases the number of eosinophils, the synthesis of IgE antibodies, leads to decreased AMP and filaggrin expression [90]. IL-4 is able to inhibit Th1 and Th17 and stimulate Th2 when directly influencing T lymphocytes [91].

Blocking of IL-4 and IL-13 can result in several adverse effects, which are observed in individuals treated with dupilumab, tralokinumab or lebrikizumab. The majority of symptoms may be attributed to the escalation of IL-17-mediated inflammation due to the inhibition of IL-4. Most information about the adverse events has been collected on dupilumab, since it is the oldest approved drug.

#### Conjunctivitis

Conjunctivitis is probably the most famous and widely discussed adverse event after the use of biologics for AD. This topic is difficult to analyze due to inconsistent nomenclature in published papers regarding the particular ocular disorders. Moreover, real-world head-to-head studies comparing the exact frequency between the drugs are sparse.

In case of dupilumab therapy, dupilumab-induced ocular surface disease (DIOSD) or more specifically, dupilumab-associated conjunctivitis (DAC), terms have been introduced [92,93]. The most important observation is that ocular-related symptoms appear particularly in patients with AD treated with dupilumab, and rarely in patients treated for other indications, e.g. asthma or eosinophilic esophagitis [93]. That could be explained by the fact that AD patients are initially prone to ocular disorders. Recurrent conjunctivitis, keratoconus and anterior subcapsular cataracts are minor Hanifin and Rajka criteria after all [94]. However, focusing on dupilumab action, since it blocks IL-4 and IL-13, it contributes to changes in the ocular surface homeostasis [92]. It has been shown that IL-13 is involved in the goblet cell proliferation and secretion of mucin MUC5AC, which provides tear film stability. Inhibition of IL-13 leads to a lower density of goblet cells and subsequently, mucin deficiency, tear film instability, resulting in ocular surface dryness [92]. Another proposed mechanism of ocular surface disorder includes the inhibition of IL-4 and IL-13, leading to a compensatory upregulation of OX40/OX40L signaling, resulting in prolonged T cell activation and persistent conjunctival inflammation [92]. The transient eosinophilia following dupilumab (described below) is another factor that is suspected to contribute to ocular disorders due to the release of cytotoxic inflammatory mediators that subsequently damage the epithelium of the conjunctiva [92]. Finally, the increased colonization by *Demodex* mites, which is known to cause blepharitis, meibomian gland dysfunction, and ocular surface inflammation, can occur in AD patients [92].

Wollenberg et al. analyzed clinical trials of tralokinumab in the search for conjunctivitis incidence. He concluded that the prevalence was higher in patients on tralokinumab than in the placebo group, but most cases were mild and subsided [95].

In the analysis performed by Stein Gold et al. the incidence of conjunctivitis was higher in lebrikizumab-treated patients compared with placebo, but the majority of cases were mild or moderate [96].

A multicenter, retrospective, observational study of 6668 adult patients with moderate-to-severe AD revealed that conjunctivitis occurred in 10.76% of dupilumab-treated patients and 12.61% of tralokinumab-treated subjects, but the difference was not statistically significant. Interestingly, symptoms occurred earlier in patients on tralokinumab, but the dupilumab-treated cases had a higher discontinuation rate [97]. In the systematic review by Alraddadi et al. the incidence of conjunctivitis in the clinical trials with dupilumab, tralokinumab and lebrikizumab was compared. There was no statistical difference in the frequency of conjunctivitis between dupilumab and the newly approved drugs [98].

Although most scientists attribute these ocular events to the blockage of IL-4/13 signaling, the exact mechanism is still uncertain. As mentioned above, it may be difficult to compare the incidence between the three main biological drugs due to the discrepancies in the nomenclature of adverse events. Certainly, factors like a history of conjunctivitis or AD severity should be taken into account [95].

#### Eosinophilia

Eosinophilia is frequently observed in subjects with AD before the treatment, due to the nature of this dermatosis, and during the therapy with dupilumab, less commonly tralokinumab and lebrikizumab. The explanation for this phenomenon is the inhibition of IL-4 in particular, and IL-13. IL-4 regulates the expression of vascular cell adhesion molecule 1 (VCAM-1). This molecule enables the migration of eosinophils to the peripheral tissues. IL-13 is also engaged in eosinophil migration. When they are inhibited, the number of eosinophils in the blood rises. A very important meta-analysis discussing this matter revealed that eosinophilia probably does not affect the treatment outcomes and does not lead to symptoms related to high eosinophil count, especially eosinophilic granulomatosis with polyangiitis, in subjects with AD [99]. An algorithm has been proposed on how to react depending on the level of eosinophilia. Considering the number of eosinophils fluctuates and usually is not associated with any symptoms, the strategy is ‘watch and wait’. Additional tests are necessary in case of symptoms or a very high number of eosinophils [100].

As for tralokinumab, in the pooled analysis of clinical trials, the number of eosinophils fluctuated throughout the observation and there were no related adverse reactions [101].

Regarding lebrikizumab, in the study by Stein Gold et al. the incidence of eosinophilia was similar in the patients receiving lebrikizumab compared to the placebo group. The overall prevalence was low, and the abnormalities were mild/moderate and did not result in drug discontinuation [96].

#### Parasite infestations

The discussion on this topic is difficult because patients infected with parasites are excluded from the clinical trials, and the diagnostic work-up of such patients often varies and is not always appropriate (adjusted to the particular parasite), which makes it difficult to assess the real incidence.

Parasite infestations may occur, particularly during treatment with dupilumab and tralokinumab. IL-4 and IL-13 are involved in the protective immune responses to helminthes [91]. They contribute to eliminating parasites by triggering the eosinophil-mediated inflammation in the intestines and stimulating mucus secretion [102]. In one of the analyses, parasitic infestations were found in 43% of reports of dupilumab therapies [102] (in the personal experience of the authors, this percentage is much lower). The information about the parasitic infestation in subjects treated with tralokinumab is scarce. In the clinical trials of tralokinumab, there was no evidence of increased infestation [101]. Regarding lebrikizumab, which also inhibits IL-13, its product characteristic claims that it is uncertain whether it affects the immune response towards parasites. In the analysis by Stein Gold et al. there was in total only one parasitic infestation, which did not lead to drug discontinuation [96].

#### Arthritis and arthralgia

Arthritis and arthralgia are other symptoms listed as side effects of dupilumab use. A specific term has even been introduced – dupilumab-associated inflammatory arthritis (DAIA). Usually, DAIA refers to seronegative arthritis, enthesitis, or joint pain. DAIA onset may vary between the patients from as early as a week up to 19 months from the first injection [103]. Its severity is also diverse but usually mild to moderate [104]. It affects women and men equally, and there is no age or anatomic localization predilection; however, usually the involvement is symmetric and generalized [103,104]. Arthritis does not seem to be associated with the history of atopy [104]. According to the literature data, the majority of patients received non-steroidal anti-inflammatory drugs and, in some cases, dupilumab was discontinued. The pathogenesis of this phenomenon is not fully understood. The idea of how to explain the development of arthritis is that inhibition of IL-4 leads to increased secretion of IL-17, which may be responsible for arthritis [103]. IL-17, on the other hand, is known to take part in seronegative arthritis, such as psoriatic or axial spondyloarthritis. IL-17 is actually a whole family of interleukins, slightly different from each other based on their structure and functions. IL-17A is involved in arthritis in several mechanisms: it is able to promote the synthesis of matrix metalloproteinases (MMP) 1, 9 and 13 which can lead to degradation of the extracellular matrix in the joint; increases the expression of the receptor NFKB ligand – RANKL by osteoblasts which leads to the activation of osteoclasts and bone degradation; lastly, IL-17A stimulates angiogenesis followed by increased blood flow and influx of inflammatory cells to the joint. IL-17F possesses similar properties; however, it is less potent [105].

#### Facial erythema

Erythema affecting the face and neck is another adverse effect of dupilumab administration, which has been reported in clinical practice but not in clinical trials. The term dupilumab-associated facial erythema (DFE) has been introduced. It appears after 11 weeks of treatment on average. In some cases, topical or systemic treatment was introduced, but in some cases, the drug was discontinued. There are several hypotheses on why DFE occurs. The first one is simply rosacea due to the inhibition of Th2-directed inflammation and promotion of Th1-dependent response. Proliferation of *Demodex* has also been suspected [106]. Indeed, the pathogenesis of rosacea involves elevated concentrations of IL-17 in serum and tissues of such patients. IL-17 is able to promote angiogenesis [107], resulting in erythema. *Demodex* mites, which are a known contributing factor in rosacea, also have an impact on IL-17 concentrations [107]. Another potential factor contributing to the erythema in the course of dupilumab treatment is alcohol intake. There have been reports of erythema occurring after alcohol ingestion, but they have been suspected to be, in fact, caused by tacrolimus ointment use – frequently applied by AD patients. However, it cannot be the sole explanation in all cases. Thus, the potential association between alcohol and dupilumab-induced erythema remains unclear. The third proposed reason for dupilumab-associated erythema is allergic contact dermatitis (ACD). Once again, it is believed that suppression of IL-4 leads to Th1 and Th17 polarization [106]. IL-17 has indeed been implicated in the pathogenesis of ACD because its elevated expression has been found in the samples from nickel-induced skin lesions as well as in the blood of allergic patients [108].

#### Neoplasms and lymphomatoid reactions

The risk of malignancies in patients with AD is uncertain, but the greatest concern remains the risk of cutaneous lymphomas (CL) [109]. Nowadays, it is believed that there is no clear association, but as early stages of AD and CL may be clinically similar, this leads to diagnostic errors from the very beginning, and is not related to drug use [45].

A relatively recent phenomenon that has been reported is dupilumab-associated lymphomatoid reaction (DALR). DALR manifests as exacerbation of preexisting condition, usually erythematous or maculopapular, pruritic, and burning lesions. In individuals affected by DALR, it is recommended to discontinue dupilumab because the lesions may eventually progress to cutaneous T-cell lymphoma. The suggested molecular explanation is that administration of dupilumab, which blocks IL-4 and 13 signaling, could potentially contribute to the progression of initially harmless lymphoid infiltrates by promoting clonal expansion of T lymphocytes, which might lead to malignant transformation [110].

Nemolizumab is the newest drug approved for AD, which blocks a different pathway than described above. It targets IL-31 receptor, and there are high hopes for it in terms of pruritus reduction, since this receptor is widely distributed, including on neurons. The most frequently reported adverse effects in patients treated with nemolizumab are AD exacerbation, nasopharyngitis, peripheral edema, and increased activity of creatinine phosphokinase [111]. An additional reported side effect is potential exacerbation of preexisting asthma [112].

### JAK inhibitors

#### Infections

Infections are the most commonly reported adverse effects of JAK inhibitor use. Considering their mode of action, it is understandable that infections may occur. As mentioned in the introduction, JAK inhibitors influence the JAK/STAT pathways and decrease the transcription of genes related to inflammatory response. They downregulate over 50 cytokines as well as growth factors, which certainly play a role in immune response [113].

The infections involve mainly the upper respiratory tract, nasopharyngitis, and *herpes simplex virus* infection [9]. There are several risk factors associated with infections during JAK inhibitor therapy: older age, disease duration, concomitant glucocorticoid therapy, and baseline lymphopenia. The risk is also dose-dependent [113].

In a recent prospective study by van der Gang et al. specifically on AD patients 12 years old or older, the incidence of skin and extracutaneous infections was higher in subjects on JAK inhibitors compared to dupilumab (hence a biological agent). The most common were herpetic infections. The authors highlighted the need to assess the history of infections when qualifying the patient for JAK inhibitors [114].

#### Dyslipidemia and weight gain

Dyslipidemia is a quite common laboratory abnormality found in subjects treated with JAK inhibitors. Hypercholesterolemia, as well as hypertriglyceridemia and increased low-density lipoprotein (LDL), may be observed, usually 6 weeks from the treatment initiation [115]. The suggested reason is down-regulation of signaling *via* IL-6 and interferons [115], alleviating their lipid-lowering properties [116]. Another postulated mechanism is that the JAK/STAT pathway is involved in the regulation of organs associated with metabolic processes, namely liver, muscles, adipose tissue, and pancreas [117]. JAK may influence hepatic synthesis and the further release of lipoproteins [116]. The third explanation, though nonspecific, is an individual susceptibility to JAK inhibitors. It has been suggested that polymorphisms in JAK-related genes may influence binding to the receptors and subsequently, the drug efficacy and adverse effects [116].

JAK inhibitors have also been reported to cause weight gain. In the randomized controlled trials, 7% of investigated patients gained weight (especially both JAK1 and 2 inhibitors) [118,119]. In the analysis by Xiong et al. such changes were noted in 5.9% of reported patients [119]. Ibba et al. suggested that leptin, also called the satiety hormone, may be involved in this complication. Patients with chronic inflammatory dermatoses tend to have increased levels of leptin, and, moreover, its activity is associated with the JAK/STAT pathway [118]. Hence, in subjects treated with JAK inhibitors, weight has to be monitored, along with lipid parameters, to early counteract increased cardiovascular risk.

#### Acne

Acne is another complication of the treatment with JAK inhibitors. The data about this side effect is very limited. Interestingly, it is reported that acne occurs more frequently after their use in subjects with AD than in patients treated for other indications [120]. However, there may be an explanation for this, namely a younger group of patients compared e.g. to rheumatic diseases [120]. A big recent meta-analysis of acne as a result of JAK inhibitor therapy performed by Chen et al. revealed that acne is more probable the longer the duration of the treatment and larger doses; moreover, pan-JAK inhibitors are associated with the lowest incidence of acne [121]. The usual severity is mild to moderate [122]. Higher rates of acne were noted in subjects treated with abrocitinib compared to upadacitinib and baricitinib [121]. The exact underlying mechanism of acne development is not known; however, several theories have been developed. First, it has been proposed that JAK inhibitors cause hyperkeratinization of the hair follicles due to inhibition of the JAK-STAT pathway, which is associated with signal transduction of the epidermal growth factor receptors [120]. The second explanation is that JAK inhibitors block the Th2-mediated inflammation, which directs the immunological processes towards Th1 and Th17, which affects the skin microbial colonization [120]. The difference between the influence of particular drugs on the acne development is explained by the different extent of inhibition of phosphorylated STAT by particular agents [123]. Contrary to what has been said, there are reports that JAK1 and JAK3 have been proven to be overexpressed in acne lesions [121], so theoretically, inhibition of JAKs should alleviate acne. Notably, besides the systemic treatment, it must be highlighted that the everyday care for AD-affected skin (emollients, vaseline) as well as topical agents (steroid or calcineurin inhibitor ointments) contribute to acne development due to their comedogenic effect [120].

### Thromboembolic complications

Concerns regarding the venous thromboembolism (VT) and major adverse cardiovascular events (MACE) have been raised after the use of another JAK inhibitor – tofacitinib – used especially for RA, but not approved for any indication described in this review paper. Afterwards, a warning was placed on every JAK inhibitor, excluding deucravacitinib. The results of subsequent studies have shown that the risk of VT/MACE is higher, the more baseline cardiovascular risk factors the patient presents. Hence, it is absolutely crucial to analyze the underlying disease for which the patient is prescribed a JAK inhibitor, its severity, and initial comorbidities. In the cohort study by Schneeweiss et al. chronic inflammatory skin diseases (psoriasis, AD, alopecia areata, vitiligo, hidradenitis suppurativa) were reported not to be associated with a higher prevalence of VT after adjusting for relevant risk factors in a representative population of patients with dermatoses [124]. In the USA, baricitinib is approved with a boxed warning for serious infections, malignancies, and thrombosis; abrocitinib and upadacitinib additionally with mortality and MACE [29,124]. In September 2024, ‘the black triangle’, a European equivalent label, was removed from the upadacitinib leaflet [125], it has also been removed from baricitinib [126]. Abrocitinib still carries that label – the product characteristics still contain information about additional monitoring regarding serious infections and VT/MACE [127].

#### Neoplasms

Since JAK inhibitors possess anti-inflammatory properties, concerns regarding the promotion of malignancies occur. In general, the incidence of neoplasms in patients receiving these drugs is low; they occur more frequently in older individuals who have been treated with immunosuppressive agents for a long time. When considering JAK inhibitors used in the treatment of AD, the risk of malignancy seems to be lower than for other drugs (tofacitinib, oral ruxolitinib) [45].

Lastly, an additional, more rarely reported side effect of JAK inhibitors, particularly upadacitinib, may be amenorrhoea. Teng et al. reported a case of a patient with amenorrhoea occurring after the introduction of this drug and resolved after its discontinuation. They suggested that the JAK-STAT pathway may therefore be involved in the signaling at the ovarian follicle function [128].

## Conclusions

The biological and small molecular treatment are relatively novel and ‘game-changing’ therapeutic options for patients with inflammatory skin diseases, which enable, in many cases, complete or almost total resolution of skin lesions. Obviously, they may exert side effects, but usually they occur infrequently and resolve after discontinuation of the treatment. In AD, most side effects—such as conjunctivitis, eosinophilia, and arthritis—are linked to IL-4 and IL-13 blockade, which may lead to increased IL-17 activity. In psoriasis, complications are more frequently associated with TNFα and IL-17 inhibition, including infections, paradoxical inflammation, and mucocutaneous candidiasis. IL-23 inhibitors, by contrast, appear to have a more favorable safety profile with fewer reported adverse effects. JAK inhibitors, used in both diseases, are associated with infections, acne, dyslipidemia, and, in some cases, cardiovascular risks, particularly in patients with pre-existing risk factors. Understanding the immunological basis of these adverse effects is crucial for personalized treatment decisions, risk stratification, and effective monitoring.

## Data Availability

Data sharing is not applicable to this article as no data were created or analysed in this research.
